# The association between posterior brain cerebral circulation calcification and coronary arteries calcification and its stroke risk in a Saudi population: a retrospective study

**DOI:** 10.1186/s43055-022-00858-1

**Published:** 2022-08-08

**Authors:** Ali M. Alqarni, Mohammed J. Alsaadi, Mohammed Fatani, Dhafer M. Alahmari, Fayka K. Abdel Azeem, Mansour J. Almalki, Abdullah Alqarni, Mazen Mohammed Abounassif, Abdulrahman M. Alfuraih

**Affiliations:** 1 Medical Imaging Department, King Saud Medical City, Riyadh, 12746 Saudi Arabia; 2 Radiology and Medical Imaging Department, College of Applied Medical Sciences, Prince Sattam Bin Abdulaziz University, Alkharj, 11942 Saudi Arabia; 3 Faculty of Medicine for Girls, Radiology and Medical Imaging Department, Al Azhar University, P.O. Box 1175, Cairo, Egypt; 4 Department of Medicine, University of Bisha, P.O. Box 61922, Bisha, Saudi Arabia

**Keywords:** Coronary arteries, Calcification, Computed tomography, Posterior brain cerebral circulation

## Abstract

**Background:**

Anterior cerebral circulation calcification and stroke occurrence association is well established in the literature. Posterior cerebral circulation calcification associated with coronary calcification and stroke incidence has not been properly investigated in Saudi Arabia. Therefore, the present study aimed to investigate the clinical relationship between posterior cerebral circulation calcification and coronary artery calcifications and to describe the association between risk factors and stroke occurrence based on computed tomography imaging.

**Results:**

A total of 101 patients were enrolled in the study. The mean age was 64.9 ± 12.4. Of the patients, 69.3 were male. Most of the patients were Saudi (72.3%), 28.6% were smokers, 36.4% were overweight, and 22.1% were obese. Exactly 61.4% had mild coronary artery calcification, 26.7% had moderate calcification, and 11.9% had severe calcification. 34.7% had an anterior stroke, and 23.8% had a posterior stroke. Posterior cerebral circulation calcification was more evident in patients with coronary artery calcification, and it increased with the severity level (*p* = 0.001). Posterior cerebral circulation calcification was significantly associated with stroke (67.4%, *p* =  < 0.001).

**Conclusion:**

Coronary artery calcification is significantly associated with posterior cerebral circulation calcification. Furthermore, stroke incidence increased with the presence of posterior cerebral circulation calcification.

## Background

Atherosclerosis is one of the underlying causes of cardiovascular events. It usually develops in many arteries (large and small arteries and those feeding the heart, brain, kidneys, and extremities), although it may occur more in some parts of the body than others. Due to aging populations, the global burden of atherosclerosis and its clinical consequences will continue to rise in the coming decades [1]. It is well known that calcium is the most abundant mineral in bones and teeth. However, with age, calcium may become deposited in various parts of the body, such as the arterial wall, which will lead to accumulation and eventually cause vascular injury, inflammation, and obstruction [2]. The deposition of atherosclerosis over time will lead to frequent vascular disease. The development of atherosclerosis can lead to major clinical conditions, such as ischemic heart disease and stroke, both of which are the top causes of global morbidity and mortality [3]. It is common in men and increases with age. Risk factors include diabetes, dyslipidemia, smoking, hypertension, chronic renal diseases, and high baseline C-reactive protein levels. Coronary artery calcification (CAC) is associated with the progress of advanced atherosclerosis. It starts with microcalcifications, which grow into larger calcium fragments, resulting in sheetlike deposits. Computed tomography intravascular imaging is a standard imaging technique used to identify CAC [4].

Intracranial artery calcification (IAC) can be located in anterior or posterior vessels, and it raises the risk of vascular events within 2 years of acute ischemic stroke [5]. IAC is linked to higher stroke rates, independent of cardiovascular comorbidity [6]. However, there is limited knowledge on IAC in vertebrobasilar vessels (posterior cerebral circulation) and its role in stroke occurrence, vascular events, or death [7]. Advances in imaging technology have improved the ability to detect and quantify atherosclerosis at all stages and in multiple different vascular beds [8]. Examining and comparing the relationships between CAC and cerebral small-vessel diseases with conventional cardiovascular risk factors is important to understand the pattern of such associations. Previous studies have investigated the correlation between anterior intracranial calcification and stroke incidence [9, 10].

Studies have also correlated CAC with anterior cerebral circulation calcification. However, little is known about posterior cerebral circulation calcifications (PCCs) and their roles in stroke occurrence, vascular events, or death [8]. Studies examining the magnitude and pattern of coexistence between subclinical cerebrovascular diseases and CAC in the general population are still limited [11]. Therefore, comprehensive knowledge of calcifications pathophysiologic mechanism, neuroimaging, and clinical features is imperative to further study possible preventive and therapeutic measures [12]. CAC may be related to PCC and stroke occurrence. Due to the limited data in the literature, the prevalence of posterior circulation stroke and its association with calcification as well as the risk factors that are prevalent in the Saudi population remain unclear. Thus, the aim of the present study investigates the association between CAC and PCCs and describes the relationship between risk factors and stroke incidence.

## Methods

### Study design and patient enrollment

This study was a retrospective and conducted at King Saud Medical City, Riyadh City, Saudi Arabia. It is one of the largest tertiary care centers in Saudi Arabia, with a total bed capacity of 1500. The institutional review board at King Saud Medical City approved the study (H1R1-09-Mar21-02). The hospital file data of 987 patients who underwent coronary and brain vascular computed tomography (CT) radiography between September 2019 and September 2020 were retrieved.

### Data collection

- Personal information (age, gender, nationality, smoking status).
- The presence of comorbidities (diabetes, hypertension, coronary heart disease), clinical presentation.
- Radiological findings (CT findings, history of stroke, stroke location, type of stroke), posterior circulation calcium, and vascular findings (vertebral arteries, basilar artery, posterior cerebral artery).

The picture archiving and communication system (PACS) was searched using “coronary calcification” as a keyword to retrieve data on patients with coronary calcification.

### Inclusion criteria

- Patient who had chest and brain CT scans between September 2019 and September 2020 (*n* = 987).
- Patient with coronary arteries calcifications.
- Patient with posterior circulation calcifications.

### Exclusion criteria

- Patient with negative CAC (*n* = 852).
- An interval of over 6 months between non-gated chest CT and brain CT (*n* = 23).
- CT brain examinations with suboptimal image quality (e.g., posterior fossa beam-hardening) (*n* = 11).

The data of *n* = 101 patients with positive CAC who underwent non-enhanced brain CT were collected for the study.

### Computed tomography (CT) examinations techniques and protocol

For non-gated chest CT, a multi-slice helical CT scanner was utilized (multi-slice 64-detector CT scanner) (Discovery CT750 HD; GE Healthcare, Chicago, IL, USA). The patients were scanned supine with their arms elevated above the head. Inspiration breathing instructions were given to reduce motion artifact (120–140 kV, Tube current–time product, 40–80 mAs depending on the patients' habitus, and 2.5 slice thickness), and 0.625-mm thin slices were used for the chest when the data were missed.

For plain brain CT examinations, the scan was performed on either a 64-detector CT scanner (Discovery CT750 HD; GE Healthcare, Chicago, IL, USA) using a conventional CT technique (120 kV, 90 mAs, and 0.5 slice thickness) or a dual-source CT scanner (Somatom Definition Flash; Siemens; Germany) using a similar conventional CT technique (120 kV, 250 mAs, and 0.5 slice thickness).

### CT examination protocol

Local CT protocols were used as follows:

- Reconstructed axial, sagittal, coronal sets of images were utilized for chest CT scan. The spiral mode was used for calcium scoring on all patients with heart rates up to 70 bpm. For patients with heart rates greater than 70 bpm, prospective sequential imaging was used instead. All scans are performed using the ALARA principle.
- Brain CT images used 5 mm axial reconstruction, 3 mm coronal, and 3 mm sagittal.

### Image analysis

**Fig. 1:**
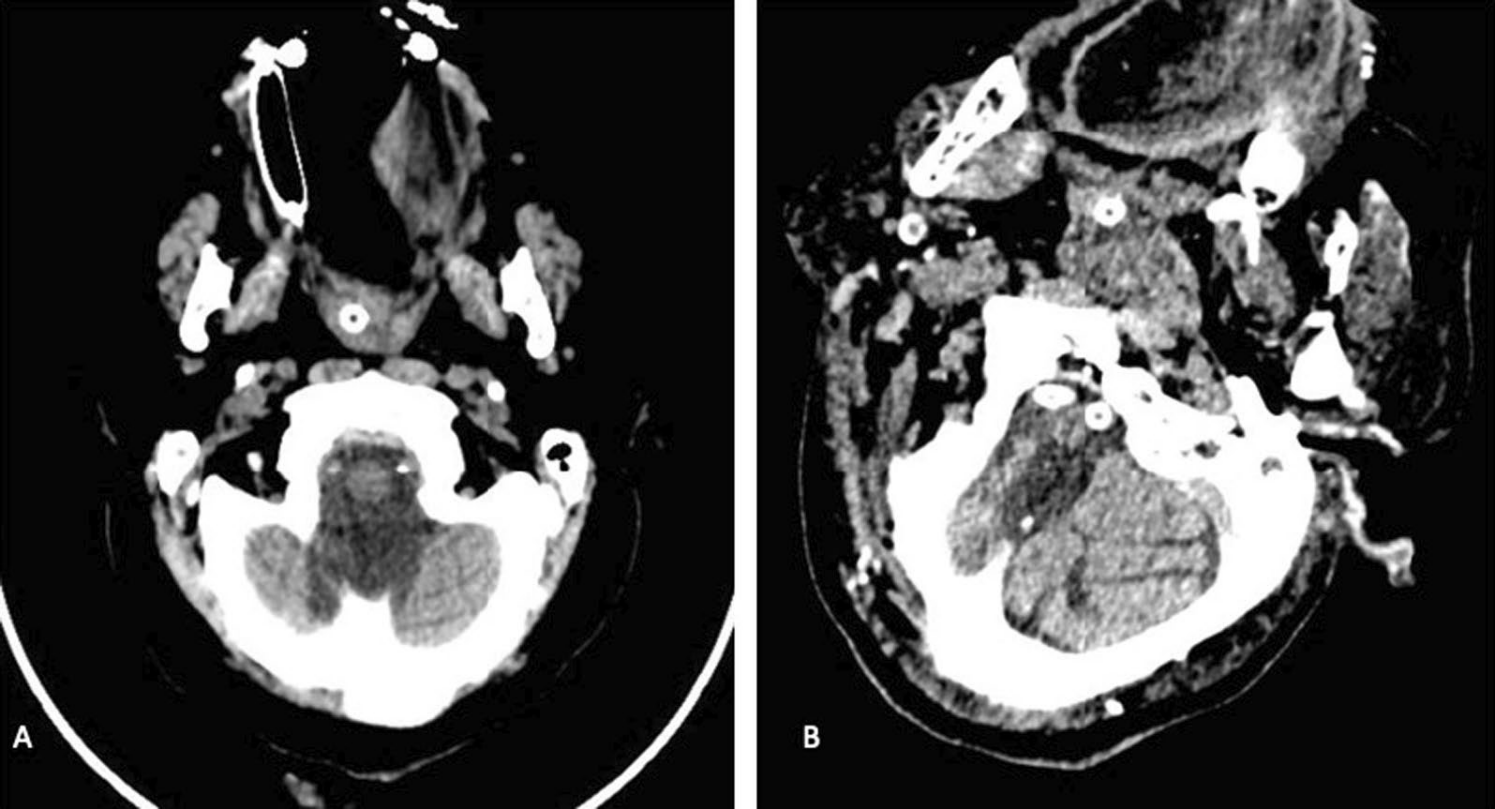
Two different patients showing posterior circulation calcification. **A** A 46-year-old male patient with a history of posterior stroke, non-enhanced CT brain of posterior fossa demonstrating dense calcifying in the left vertebral artery. **B** A 55-year-old male patient with a history of stroke, non-enhanced CT brain of posterior fossa showing a dense calcification in bilateral vertebral arteries

All brain images were assessed by a neuroradiologist consultant with over 20 years of experience. A set of thin-slice brain images of all included patients were reviewed. The posterior calcification was categorized according to the presence or absence of calcification in the following vascular beds: right vertebral artery, left vertebral artery, and basilar artery (Fig. 1).

**Fig. 2:**
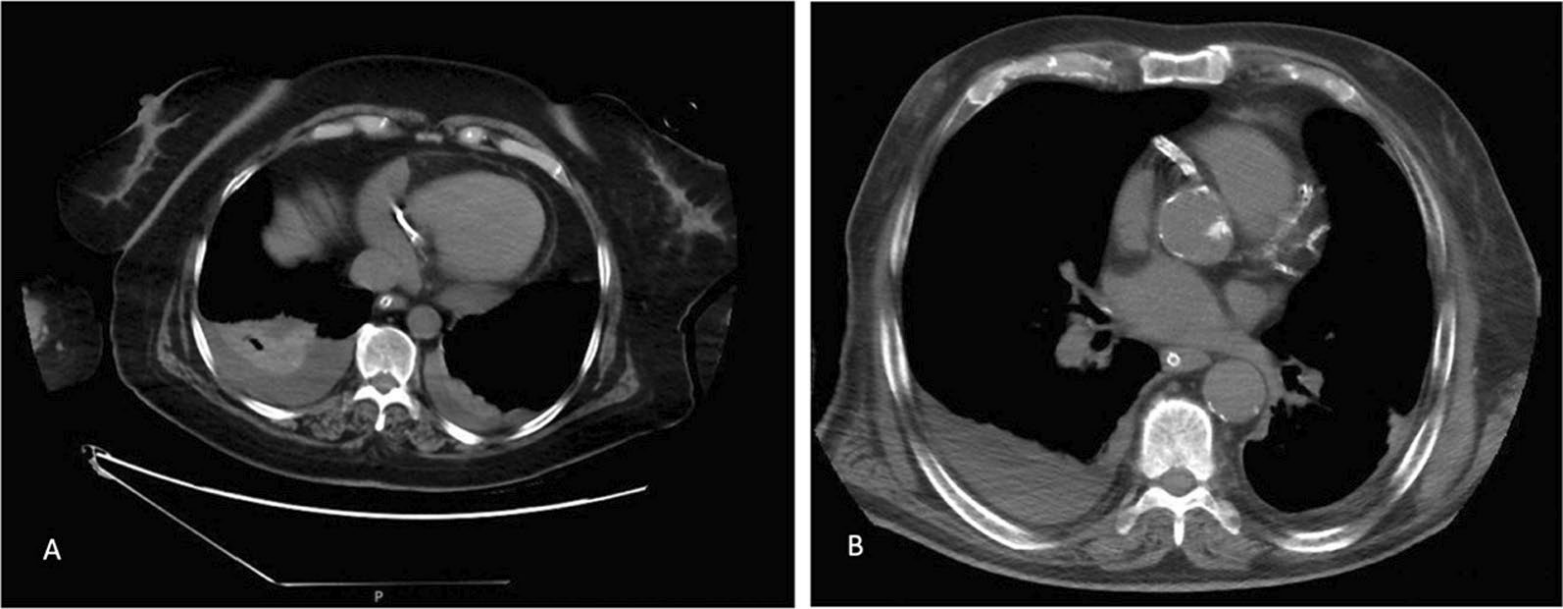
Two different patients showing coronary arteries calcification. **A** A 59-year-old male with a history of heart attack, non-contrast CT image showing severe, dense coronary calcifications involving the RCA with mild bilateral plural effusion more in the right side with associated atelectatic changes. **B** A 63-year-old man presented with a history of HTN, smoking and previous stroke, non-contrast CT image showing moderate-to-severe, dense coronary calcifications in the aortic route and involving the left system (LCX and LAD) as well as RCA, mild right-sided plural effusion also noted

All chest images were reviewed by radiologists with a subspecialty in thoracic imaging and over 13 years of experience. The positive CAC was assessed visually, categorizing the calcification as mild, moderate, or severe based on the guidelines suggested [13] (Fig. 2**)**.

### Coronary arteries calcification scoring

The Agatston scoring system is the most quantitative scoring system for reporting the calcification of coronary arteries. Nevertheless, this technique requires special software and hardware to implement as well as standard scanning parameters, such as a small field of view (FOV) and a radiation dose necessary for a higher resolution. Therefore, the Agatston scoring system was not used. Published guidelines recommend a visual assessment and report on coronary artery calcification [13]. A visual assessment system of calcification can determine an absence of calcification, mild calcification, moderate calcification, and severe calcification. The visual estimation method has the advantage of feasibility and straightforward implementation [13].

### Posterior circulation calcification scoring

To the best of our knowledge, there is no existing method for evaluating and scoring intracranial arterial calcification. A recent study reviewed the literature and discussed the assessment of intracranial calcification. The author confirmed the lack of an available method and recommended further trials to develop a consistent calcification scoring tool [14].

### Statistical analysis

Descriptive and inferential statistics were used to assess the study variables. All analyses were done using SPSS version 27 (Armonk, NY: IBM Corp). A binomial logistic regression was conducted to evaluate the association between the incidence of stroke and the relevant independent variables based on previously described methods [15]. The relevant independent variables were tested for associations with stroke using the chi-square test for nominal or ordinal variables and a univariate logistic regression for continuous variables. Variables with *p*-values < 0.25 were subjected to a multivariate logistic regression using the backward stepwise approach. The final model included statistically significant (*p* < 0.05) independent variables. After removing each insignificant variable, we monitored the model fit using the log-likelihood ratio to ensure it did not significantly influence the model. The independent variables included age, gender, BMI, hypertension, diabetes, smoking, presence of a brain lesion, headache, dizziness, neurological symptoms, loss of consciousness, CAC severity, and the presence of PCC. An alpha value of 0.05 was considered significant across all tests.

## Results

A total of 101 patients participated in the study. The mean age (SD) was 64.9 ± 12.4 years. More than two-thirds of patients (69.3%) were male. Most of the patients were Saudi (72.3%). More than one-third (36.4%) were overweight, while 22.1% were obese. Almost two-thirds of patients (61.4%) had mild CAC, 26.7% had moderate calcification, and 11.9% had severe calcification. Stroke location was in the anterior cranial region in 34.7% of patients, while it was in the posterior region in 23.8%. Almost two-thirds of patients were diabetic (63.6%), 76.6% were hypertensive, 29.9% had ischemic heart disease, and 28.6% were smokers, as shown in Tables 1 and 2.

**Table 1:** Demographic data of patients with coronary artery calcification (*n* = 101)

Personal characteristics	No.	%
*Age-groups*		
< 60 years	33	32.7
60–70 years	35	34.7
> 70 years	33	32.7
Mean ± SD	64.9 ± 12.4 years	
*Gender*		
Female	31	30.7
Male	70	69.3
*Nationality*		
Saudi	73	72.3
Non-Saudi	28	27.7
*Smoking status*		
Non-smoker	73	72.3
Smoker	28	27.7
*Body mass index*		
< 25 kg/m^2^	37	36.6
25–29.9 kg/m^2^	38	37.6
30 + kg/m^2^	26	25.7
*Coronary artery calcification severity*		
Mild	62	61.4
Moderate	27	26.7
Severe	12	11.9
*Stroke*		
No	54	53.5
Yes	47	46.5
*Diabetes mellitus*		
No	44	43.6
Yes	57	56.4
*Hypertension*		
No	39	38.6
Yes	62	61.4
*Ischemic heart disease*		
No	74	73.3
Yes	27	26.7

**Table 2:** Clinical and radiological findings of patients with coronary artery calcification

Clinical/radiological findings	No	%
*Clinical findings*		
Dizziness	9	8.9
Headache	18	17.8
Loss of consciousness	36	35.6
Neurological symptoms	35	34.7
Brain lesions	7	6.9
*Comorbidity*		
Diabetes	57	56.4
Hypertension	62	61.4
*Location of stroke*		
Anterior circulation	35	34.7
Posterior circulation	24	23.8
*Location of calcification*		
Posterior calcification	43	42.6
Left vertebral artery	39	38.6
Right vertebral artery	28	27.7
Basilar artery	4	4.0

The most common clinical and radiological findings among coronary artery calcification patients were loss of consciousness (35.6%) and headache (17.8%). Diabetes and hypertension were the most common associated morbidities among coronary artery calcification cases (63.6% and 76.6%, respectively). Moreover, 23.8% of patients had a stroke in the posterior circulation, and 34.7% had a stroke in the anterior circulation. Calcification was primarily in the left (37.6%) or right (27.7%) vertebral artery. Basilar artery calcification was present in 4% of cases, as shown in Table 2.

**Fig. 3:**
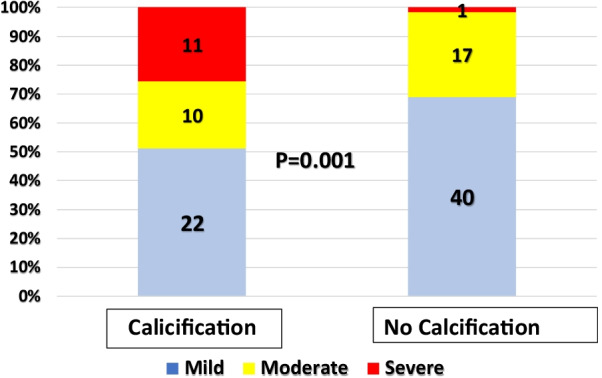
Grades of coronary artery calcification according to presence of posterior circulation calcification

Figure 3 compares the existence of PCC according to the grade of coronary artery calcification. The occurrence of PCC is significantly associated with severe CAC (*p* = 0.001).

Table 3 shows that the presence of posterior circulation calcification was significantly more associated with patients with stroke than with those without stroke (67.4% and 32.6%, respectively, *p* =  < 0.001).

**Table 3:** Association of posterior circulation calcification and stroke occurrence

Posterior circulation calcification	Occurrence of stroke				*p* value
	No. (n = 54)		Yes (n = 47)		
	No.	%	No	%	
Absent	40	69.0	18	31.0	
Present	14	32.6	29	67.4	< 0.001

PCC was prevalent in 43 CAC patients (42.6%). The chi-square test of proportions showed that 29 patients with stroke also had PCC compared with 40 patients with no stroke and negative PCC, a difference in proportions of *p* < 0.001. CAC severity was not significantly associated with higher stroke proportions (*p* = 0.106). However, patients with more severe CAC had higher proportions of PCC (*p* = 0.001; Table 4).

**Table 4:** Proportions of patients with PCC according to coronary arteries calcifications (CAC) severity

CAC severity	Posterior circulatory calcification			
	No	%	Yes	%
Mild	40	64.5	22	35.5
Moderate	17	63.0	10	37.0
Severe	1	8.3	11	91.7

*p* = 0.001

Table 5 presents the variables evaluated using a univariate logistic regression. In the stepwise approach using these variables, PCC was the only remaining significant variable with an odds ratio [95% CI] of 4.6 [1.97, 10.73].

**Table 5:** Univariate logistic regression of the predictive variables

	Odds ratio	[95% CI]	*p* value
Age	1.01	[0.98, 1.04]	0.329
Male	1.62	[0.69, 2.79]	0.267
BMI	1.07	[0.99, 1.15]	0.064
Hypertension	1.68	[0.56, 5.09]	0.354
Diabetes	1.25	[0.49, 3.24]	0.632
Smoking	1.50	[0.55, 4.05]	0.424
Brain lesion	1.58	[0.33, 7.46]	0.562
Headache	0.26	[0.08, 0.87]	0.091
Loss of consciousness	0.87	[0.38, 1.98]	0.754
Coronary arteries calcifications severity	1.68	[0.94, 2.99]	0.078
PCC	**4.60**	[1.97, 10.73]	** < 0.001**

*p*<0.05 is shown in bold

## Discussion

This study aims to explore the association of CAC with the coexistence of PCC and stroke incidence in the Saudi population. This association has not been extensively investigated in Saudi Arabia. The results demonstrated that 42.6% of patients with CAC had PCC. Our analysis indicates that 29 patients with stroke had calcifications in the posterior circulation, indicating that calcifications in the posterior brain cerebral circulation are strongly related to stroke occurrence. Therefore, PCCs should be identified during routine brain CT scans.

CAC severity was not significantly associated with higher stroke proportions (*p* = 0.106). Moreover, severe CAC grades were significantly higher in patients with posterior brain cerebral calcification (*p* = 0.001). Calcifications were more prevalent in the left vertebral artery (*p* = 0.001). However, there was no significant difference among those with right vertebral or basilar artery calcification. Further studies are recommended to investigate the location and quantification of calcifications in each vessel.

Our study suggested that patients with CAC will likely develop PCC with an odds ratio [95% CI] of 4.6 [1.97, 10.73]. The occurrence of severe grades of CAC was significantly higher among those with dizziness symptoms (*p* = 0.027). This occurrence was not evident among patients with headaches, loss of consciousness, diabetes, hypertension, stroke, and anterior or posterior stroke. The supplementary section provides a comprehensive analysis of the correlations of symptoms and risk factors with PCC and CAC calcification.

Risk factors, such as smoking status, diabetes, hypertension, clinical symptoms, and their correlation with CAC, PCC, and stroke were also investigated. Our study revealed a significant association between CAC grade and PCC (*p* = 0.001). Patients showed an increasing prevalence of PCC with increasing CAC severity. This finding is in accordance with those reported by several studies [7, 16]. Grades of patients’ CAC did not differ significantly in patients who smoked or had diabetes, hypertension, or stroke. The relatively small sample could have caused the inconsistency with the results reported in the literature. The probability of brain stroke incidence was statistically significant among patients with diabetes (*p* = 0.001).

The findings of the present study showed that the incidence of stroke was significantly associated with PCC. Nevertheless, the association between PCC and stroke remains a controversial issue. Studies have investigated the cardiovascular risk factors associated with the existence of PCC and found that hypertension, diabetes mellitus, and smoking are associated [17].

The existence and severity of atherosclerosis in diverse arterial beds may be greater in some populations than in others, with likely implications for causes of vascular death [18]. Differences in methodology, study sample size, and genetic and environmental factors may explain the contradictions found in the literature. In this study, 72% of the sample were Saudis. Our findings cannot be generalized due to the small sample size, and further studies are required to investigate this topic nationally. The appropriate interpretation of clinical, laboratory, and imaging findings is crucial for diagnosing ischemic stroke caused by atherosclerosis in the vertebral artery or intracranial arteries. Computed tomography angiography is an excellent tool for diagnosing intracranial artery disease [19].

This study has multiple potential limitations. First, our study data were collected from one center in Riyadh. Thus, more data from different locations across the country are required to generalize the results. Second, the number of patients who had CT in the past year was low due to the COVID-19 pandemic. Third, PCC was underdiagnosed in our center, and not all patients with brain CTs had a full description of the presence of PCC in the radiology report. Moreover, the use of an automated quantitative method of intracranial calcification regardless of the extent and thickness of calcification in the vascular bed is essential for specifying the number of lesions and augmentations of calcification.

Studies are needed to investigate the correlations between a higher susceptibility of left vertebral artery calcification and common congenital variation of vertebral artery dominance. Moreover, more studies should assess intracranial calcification with a quantitative grading score to precisely appraise the correlations and associated risk factors.

In conclusion, our findings strongly suggest that severe CAC is significantly associated with PCCs. Furthermore, the occurrence of stroke due to posterior circulation calcification is significant, and the presence of calcification in the posterior circulation could be a predictor of stroke and require careful evaluation during computed tomography brain scans.

## Data Availability

All the datasets used and analyzed in this study are available with the corresponding author on reasonable request.

## Abbreviations

CAC: Coronary artery calcification
PCCs: Posterior circulation calcifications
IAC: Intracranial artery calcification
CT: Computed tomography
PACS: Picture archiving and communication system
FOV: Field of view
